# Evaluation of 30 DNA damage response and 6 mismatch repair gene mutations as biomarkers for immunotherapy outcomes across multiple solid tumor types

**DOI:** 10.20892/j.issn.2095-3941.2020.0351

**Published:** 2021-05-07

**Authors:** Zhe Gong, Yue Yang, Jieyun Zhang, Weijian Guo

**Affiliations:** 1 Department of Medical Oncology, Fudan University Shanghai Cancer Center, Shanghai 200032, China; 2 Department of Oncology, Shanghai Medical College, Fudan University, Shanghai 200032, China

**Keywords:** Immune checkpoint inhibitor therapy, prediction of efficacy, tumor mutation burden, mismatch repair deficiency, DNA damage response genes

## Abstract

**Objective:**

DNA damage response (DDR) genes have low mutation rates, which may restrict their clinical applications in predicting the outcomes of immune checkpoint inhibitor (ICI) treatment. Thus, a systemic analysis of multiple DDR genes is needed to identify potential biomarkers of ICI efficacy.

**Methods:**

A total of 39,631 patients with mutation data were selected from the cBioPortal database. A total of 155 patients with mutation data were obtained from the Fudan University Shanghai Cancer Center (FUSCC). A total of 1,660 patients from the MSK-IMPACT cohort who underwent ICI treatment were selected for survival analysis. A total of 249 patients who underwent ICI treatment from the Dana-Farber Cancer Institute (DFCI) cohort were obtained from a published dataset. The Cancer Genome Atlas (TCGA) level 3 RNA-Seq version 2 RSEM data for gastric cancer were downloaded from cBioPortal.

**Results:**

Six MMR and 30 DDR genes were included in this study. Six MMR and 20 DDR gene mutations were found to predict the therapeutic efficacy of ICI, and most of them predicted the therapeutic efficacy of ICI, in a manner dependent on TMB, except for 4 combined DDR gene mutations, which were associated with the therapeutic efficacy of ICI independently of the TMB. Single MMR/DDR genes showed low mutation rates; however, the mutation rate of all the MMR/DDR genes associated with the therapeutic efficacy of ICI was relatively high, reaching 10%–30% in several cancer types.

**Conclusions:**

Coanalysis of multiple MMR/DDR mutations aids in selecting patients who are potential candidates for immunotherapy.

## Introduction

Cancer is recognized as a global health problem and is expected to become the leading cause of death worldwide^1^. Over the past few years, immune checkpoint inhibitors, including antibodies targeting programmed death receptor 1 (PD-1) or its ligand (PD-L1), and cytotoxic T lymphocyte-associated protein 4 (CLTA4) have shown impressive clinical efficacy against several types of solid tumors^2,3^. Despite the clinical efficacy of ICIs, most patients do not respond well to them. Therefore, methods for selecting patients who respond well to ICIs remain to be addressed.

A series of biomarkers have been approved for clinical prediction of the efficacy of ICIs; these include PD-L1^4^, as well as mismatch repair deficiency (dMMR)^5^, immune cell infiltration^6^, and TMB^7^. MMR gene deficiencies, which give rise to genomic instability, can cause mutation accumulation or microsatellite instability (MSI). Thus, patients with dMMR tumors are more likely to have high TMB and to have better responses to ICI. Although dMMR has been widely recognized and used clinically, its application is limited to certain cancers, because of the low mutation rate in major cancers. Hence, some researchers are investigating DDR genes^8^. Deficiencies in DDR genes are also associated with functional losses in sensing and repairing DNA damage, thus leading to high PD-1/PD-L1 expression, MSI, and increases in the TMB, neoantigen load, and immune response^9–12^. A small retrospective study has found that mutations in DDR genes are associated with better responses to PD-1/PD-L1 inhibitors in urothelial cancer, but large sample studies in diverse cancer types remain lacking^13^. A previous study has shown that deficiencies in 2 DDR genes, *POLD1*/*POLE*, are associated with increased TMB and have the ability to predict ICI efficacy in diverse cancers^14^.

However, individual DDR genes have low mutation rates, thereby potentially restricting clinical applications. Consequently, a systemic analysis of multiple DDR genes is needed to identify potential biomarkers of ICI efficacy. Therefore, we performed this study to provide more efficacy predictors for clinical ICI treatment, by analyzing the associations among multiple MMR and DDR gene mutations and TMB and the therapeutic efficacy of ICI.

## Materials and methods

### Study population

A total of 39,631 patients with mutation data were selected from the cBioPortal database (https://www.cbioportal.org). A total of 155 patients with mutation data were obtained from FUSCC. All nonsynonymous mutations, including nonsense, nonstop, splice site, missense, frame-shift, and translation start site alterations in MMR and DDR genes, were included in our study. RNA-Seq data from gastric cancer in The Cancer Genome Atlas (TCGA) were also obtained from the cBioPortal database for immune cell analysis. The MSI status of TCGA patients was obtained from the Genomic Data Commons Data Portal (https://portal.gdc.cancer.gov/). A total of 1,660 patients from the MSK-IMPACT cohort who underwent ICI treatment^15^ were selected for survival analysis. A total of 249 patients who underwent ICI treatment from the Dana-Farber Cancer Institute (DFCI) cohort were obtained from a published dataset^16^ for efficacy and survival analyses.

All patients provided written informed consent for their tissues to be used in this work, and the use of patient tissue samples and the study protocol were approved by the FUSCC ethics committee.

### Whole-exome sequencing

To obtain mutation data from the FUSCC cohort, we treated genomic DNA samples of gastric cancer tissue with a SeqCap EZ capture kit (Roche) to construct whole-exome sequencing libraries^17^. A Qubit 2.0 fluorometer (Life Technologies) and a NanoDrop 2000 spectrophotometer (Thermo) were used to determine the DNA concentration and quality. A SeqCap EZ capture kit (Roche) was used to prepare the whole-exome sequencing libraries according to the protocol recommended by Illumina. Furthermore, we used VARSCAN software to identify somatic single-nucleotide and indel mutations. MSI sensor^18^ software was used to calculate the MSI score for each patient in the FUSCC cohort, and patients with MSI scores >18 were placed in the MSI-H group.

### Bioinformatic analysis

TCGA level 3 RNA-Seq version 2 RNA-Seq by Expectation Maximization (RSEM) data were downloaded from cBioPortal. Gene expression was estimated with the RSEM method. The CIBERSORT algorithm was used to estimate the absolute scores of 22 immune cells for each sample in TCGA cohort.

### Statistical analysis

All analyses were conducted in R software version 3.6.0 (https://www.r-project.org/) and SPSS 20.0 (SPSS Inc., Chicago, IL, USA). Kaplan–Meier curves and log-rank tests were used to evaluate the relationships between the different subgroups and overall survival (OS). Univariate and multivariate Cox regression analyses were performed to identify independent prognostic factors. Student’s *t* tests were used to compare variables between groups. Two-tailed *P* < 0.05 was considered statistically significant, and, because of multiple hypothesis testing, the log-rank test significance values were set and interpreted at *P* = 0.0016 by using the Bonferroni method for multiple comparison adjustment (*P* = 0.05/30).

### Subgroup definitions for the MSKCC-IMPACT and DFCI cohorts

The MMR mutant subgroup was defined as patients who had at least one MMR gene mutation and TMB >20 mutations/MB. The remaining patients who had at least one mutation in any DDR gene except *TP53* were placed in the DDR mutant subgroup. Then the patients who had *TP53* mutations were placed in the *TP53* mutation subgroup, and the remaining patients were placed in the wild-type subgroup.

## Results

### Associations among MMR gene mutations, MSI, and TMB in TCGA colorectal and gastric cancer and the FUSCC gastric cancer cohorts

The median TMBs of the entire TCGA colorectal and gastric cancer and the FUSCC gastric cancer cohorts were 4.32, 4.47, and 2.84 mutations/MB, respectively, whereas the median TMBs of patients diagnosed with MSI-H in these cohorts were 45.39, 45.34, and 77.01 mutations/MB, respectively. The chi-square test indicated that patients diagnosed with MSI-H tended to have TMB >20 mutations/MB (**Supplementary Figure S1A, S1B**, *P* < 0.05). We focused on 6 MMR genes: *MLH1*, *MSH2*, *MSH3*, *MSH6*, *PMS1*, and *PMS2*. The median TMB values of patients in TCGA colorectal and gastric cancer cohorts with and without MMR gene mutations are listed in **Supplementary Table S1**, and patients with any MMR gene mutations tended to have high TMB. The chi-square test indicated that patients with MSI-H tended to have MMR gene mutations (**Supplementary Figure S1C, S1D**, *P* < 0.05). Moreover, in TCGA colorectal and gastric cancer and the FUSCC gastric cancer cohorts, 54.55%, 77.27%, and 63.64% of patients with MMR gene mutations were diagnosed with MSI-H, respectively, and 80.00%, 69.86%, and 53.33% of patients diagnosed with MSI-H had at least one mutation in MMR genes.

### Associations among DDR gene mutations, MSI status, and TMB in TCGA colorectal and gastric cancer cohorts

The DDR pathway comprises many components, such as MMR, base excision repair, checkpoint factors, Fanconi anemia, homologous recombination repair, nucleotide excision repair, nonhomologous end-joining, and DNA translesion synthesis^8^. On the basis of previous studies^8,19^, we focused on 30 DDR genes: *ATM*, *ATR*, *BLM*, *BRCA1*, *BRCA2*, *BRIP1*, *CHEK2*, *ERCC2*, *ERCC3*, *ERCC4*, *ERCC5*, *FANCA*, *FANCC*, *MDC1*, *MUTYH*, *NBN*, *PALB2*, *PARP1*, *POLD1*, *POLE*, *PTEN*, *RAD50*, *RAD51*, *RAD51B*, *RAD51C*, *RAD51D*, *RAD52*, *RAD54L*, *RECQL4*, and *TP53*. The median TMB of patients in TCGA colorectal and gastric cancer cohorts with and without DDR gene mutations is listed in **Supplementary Tables S2 and S3**, respectively, and patients in TCGA colorectal and gastric cancer cohorts with mutations in any DDR gene except *TP53* tended to have higher TMB. We further analyzed the association between DDR gene mutations and MSI status. Three patients in TCGA colorectal cancer cohort were diagnosed with MSI-H and had no MMR gene mutations, whereas all of them (100%) had at least one mutation in a DDR gene (except *TP53*). Moreover, 22 patients in TCGA gastric cancer cohort were diagnosed with MSI-H and had no MMR gene mutations, whereas 19 (86.36%) had at least one mutation in any DDR gene (except *TP53*). Thus, our study indicated that DDR gene mutations might also result in MSI-H.

### The expression levels of PARP1 and PDL1 in groups with different DDR gene mutations and MSI and TMB status

We further analyzed the expression levels of PARP1 and PDL1 in groups with different DDR gene mutations and MSI and TMB status, by using TCGA gastric cancer cohort. The Oncoprint^20^ image (**Supplementary Figure S1E**) indicated no correlation between the mutational status of DDR/MMR genes and the expression levels of PARP1/PDL1. Student’s *t* test also indicated that the differences in the expression levels of PARP1 and PDL1 in groups with different MSI/TMB were not statistically significant.

### The association between MMR/DDR gene mutations and TMB in the MSK-IMPACT and DFCI cohorts

We also analyzed the association between MMR/DDR gene mutations and TMB in the MSK-IMPACT and DFCI cohorts. The compositions of different types of cancers in the MSK-IMPACT and DFCI cohorts are presented in **Supplementary Figure S2A and S2B**, respectively. As shown in **Supplementary Table S4**, all groups with MMR/DDR mutations except those in *TP53* had a significantly higher median TMB than the wild-type groups. Patients with at least one MMR gene mutation had a median TMB of 26.56 mutations/MB and 31.76 mutations/MB in the MSK-IMPACT and DFCI cohorts, respectively.

### High TMB is associated with better therapeutic efficacy of ICI and more tumor-infiltrating CD8^+^ T cells

**Figure 1:**
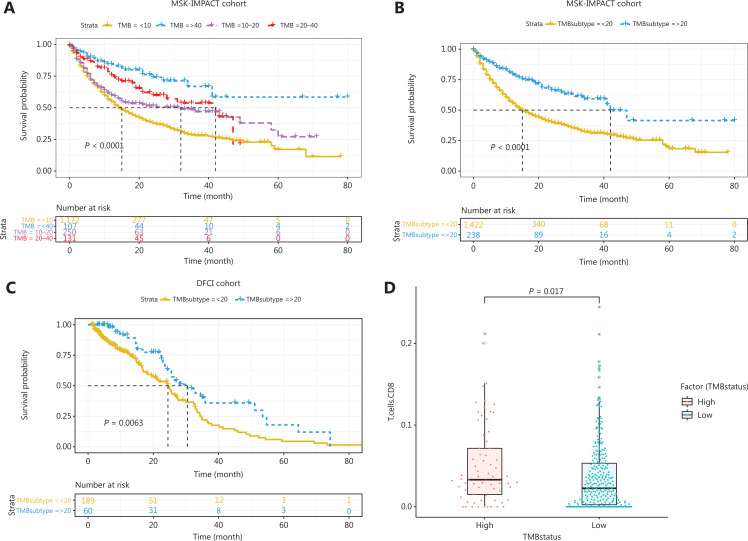
Associations between TMB and ICI therapy outcomes. (A) Kaplan–Meier analysis of patients with different TMB in MSK-IMPACT cohort. (B) Kaplan–Meier analysis showed that patients in the MSK-IMPACT cohort with TMB >20 mutations/MB showed significantly better efficacy than patients with TMB <20 mutations/MB. (C) Kaplan–Meier analysis showed that patients in the DFCI cohort with TMB >20 mutations/MB showed significantly better efficacy than patients with TMB <20 mutations/MB. (D) Students’ *t* test indicated that high TMB was associated with statistically significantly higher absolute scores of tumor-infiltrating CD8^+^ T cells.

We analyzed outcomes in patients with different TMBs and found that higher TMB was directly associated with better therapeutic efficacy of ICI (**Figure 1A**, *P* < 0.05). Notably, patients with a TMB >20 mutations/MB had significantly better efficacy than patients with a TMB <20 mutations/MB in the MSK-IMPACT and DFCI cohorts (**Figure 1B, 1C**, *P* < 0.05). Furthermore, we explored the associations between TMB and the absolute scores of tumor-infiltrating CD8^+^ T cells in TCGA gastric cancer cohort to determine the mechanism through which TMB affects the therapeutic efficacy of ICI. A total of 395 patients with available RNA-Seq data were placed in the TMB-H and TMB-L groups according to the cutoff TMB value of 20 mutations/MB. Student’s *t* test analysis revealed that the TMB-H group had significantly higher absolute scores of tumor-infiltrating CD8^+^ T cells (**Figure 1D**, *P* < 0.05).

### Associations between MMR/DDR gene mutations and the therapeutic efficacy of ICI

**Figure 2:**
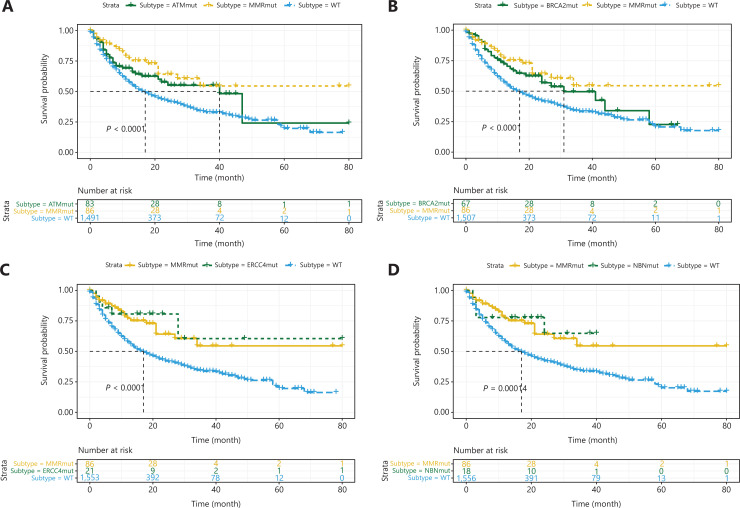
Associations between *ATM*, *BRCA2*, *ERCC4*, and *NBN* mutations and ICI therapy outcomes. Kaplan–Meier analysis of patients with and without (A) *ATM*, (B) *BRCA2*, (C) *ERCC4*, and (D) *NBN* mutations.

**Figure 3:**
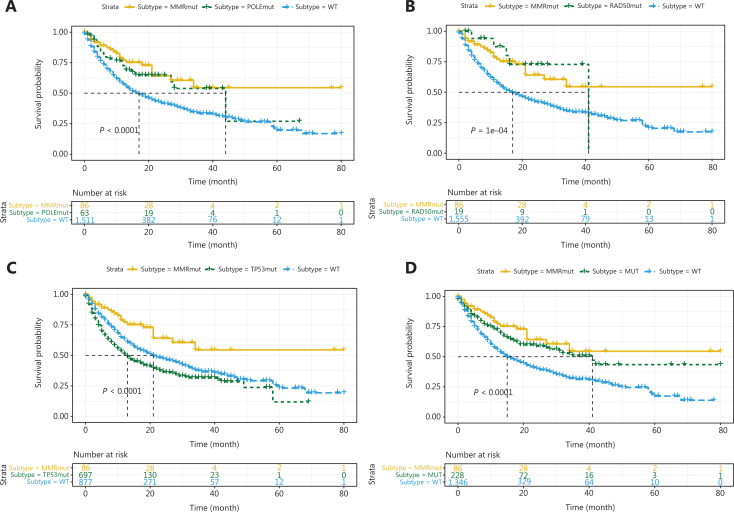
Associations between *POLE*, *RAD50*, *TP53*, 14 combined DDR mutations and ICI therapy outcomes. Kaplan–Meier analysis of patients with or without (A) *POLE*, (B) *RAD50*, and (C) *TP53* mutations. (D) Kaplan–Meier analysis of patients with or without 14 combined DDR gene mutations.

**Figure 4:**
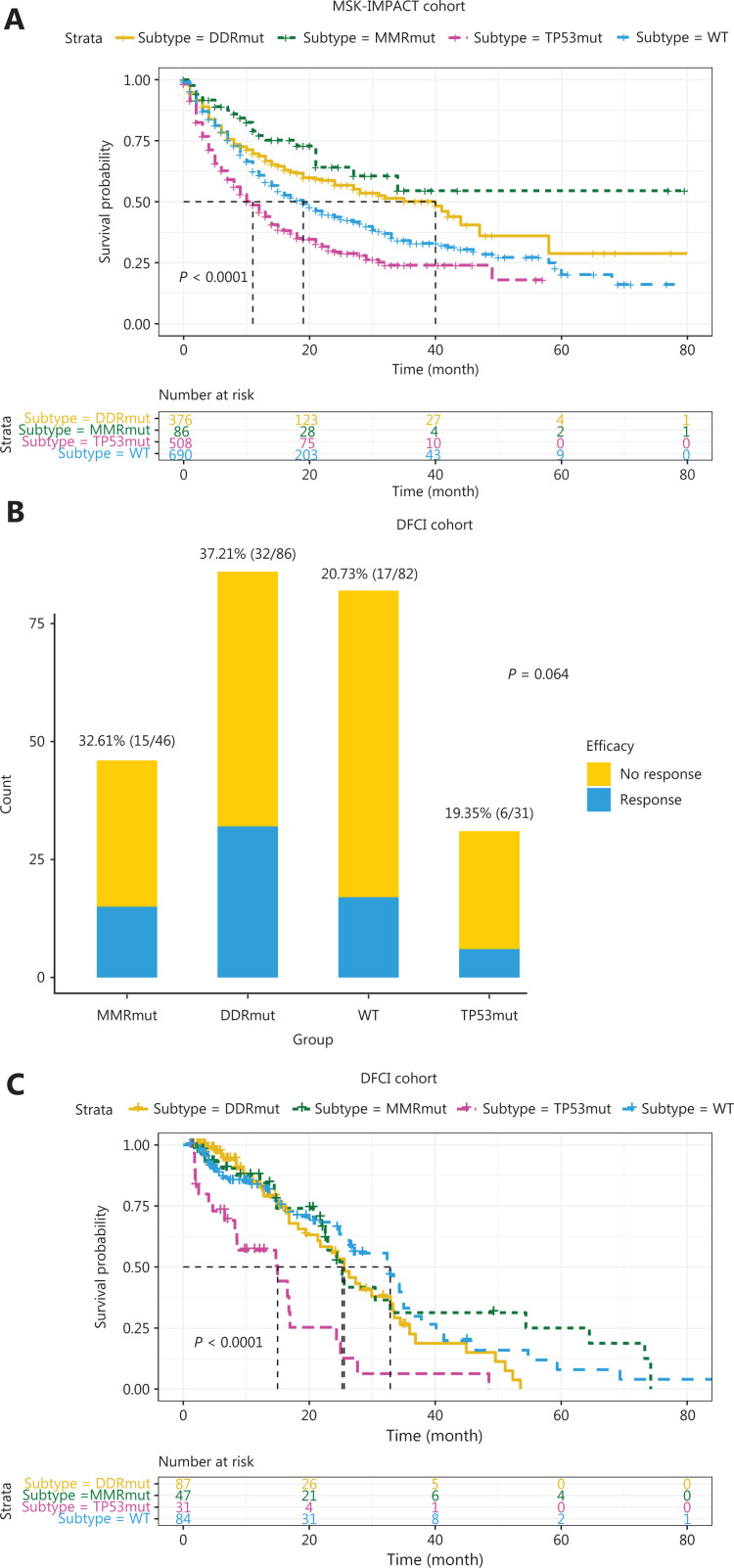
Associations between MMR/DDR mutations and ICI therapy outcomes. (A) Kaplan–Meier analysis of MMR mutant, DDR mutant, MMR/DDR wild type, and *TP53* mutant subgroups in the MSK-IMPACT cohort. (B) ORR and (C) Kaplan–Meier analysis of MMR mutant, DDR mutant, MMR/DDR wild type, and *TP53* mutant subgroups in the DFCI cohort.

As mentioned above, patients with MMR gene mutations tend to have higher TMB. MMR gene mutations cause genomic instability and a loss of the ability to repair DNA mismatches, thus resulting in many gene mutations, including DDR gene mutations, and high TMB. These patients are sensitive to immune therapy because of the high TMB caused by MMR gene mutations, and their responses may not be substantially affected by DDR gene mutations. Therefore, according to a previous study^21^ and the results discussed above, patients in the MSK-IMPACT cohort who had at least one MMR gene mutation and TMB >20 mutations/MB were placed in the MMR mutant subgroup. Log-rank tests were performed on the data for the remaining patients to identify the outcomes predictive of DDR gene mutations. *ATM*, *BRCA2*, *ERCC4*, *NBN*, *POLE*, and *RAD50* mutations were associated with favorable outcomes (**Figures 2A–2D, 3A and 3B**, all *P* < 0.001), and *TP53* mutations were associated with unfavorable outcomes (**Figure 3C**, *P* < 0.001). In addition, patients with DDR gene mutations in *ATR*, *BLM*, *BRIP1*, *CHEK2*, *ERCC2*, *ERCC3*, *ERCC5*, *FANCA*, *FANCC*, *PARP1*, *POLD1*, *RAD51*, *RAD51B*, and *RAD51C* had significantly longer median survival than other patients, although this difference did not reach statistical significance because of the small number of mutant patients. Therefore, we combined the remaining 14 DDR gene mutations and found that patients in the mutation group had better outcomes (**Figure 3D**, *P* < 0.001). Thus, among the entire MSK-IMPACT cohort, we divided patients were into 4 subgroups: MMR mutant, DDR mutant (with at least one mutation in the 21 DDR genes except *TP53*), MMR/DDR wild type, and *TP53* mutant. Kaplan–Meier curve analysis was performed, and the MMR and DDR mutant subgroups were found to be associated with better outcomes, whereas the MMR/DDR wild-type and *TP53* mutant subgroups were associated with poorer outcomes (**Figure 4A**, *P* < 0.001).

Thus, we found that MMR and 20 DDR gene mutations were associated with better OS in patients with ICI treatment. However, because of the absence of data on patient clinical response status in the MSK-IMPACT cohort, determining whether these gene mutations are associated with the therapeutic efficacy of ICI or patient prognosis is difficult. Therefore, we validated our findings in the DFCI cohort. Patients in DFCI were divided into 4 subgroups (MMR mutant, DDR mutant, MMR/DDR wild type, and *TP53* mutant), on the basis of the criteria described above. As shown in **Figure 4B**, patients in the MMR mutant and DDR mutant subgroups had higher objective response rates (ORRs). Kaplan–Meier curves were generated, and the *TP53* mutant subgroup showed an association with poorer outcomes, whereas patients in the MMR mutant, DDR mutant and MMR/DDR wild-type subgroups showed little difference in OS (**Figure 4C**, *P* < 0.05). These results indicated that MMR and DDR gene mutations, except those in *TP53*, were predictive factors rather than prognostic factors for ICI therapy.

### Most MMR/DDR gene mutations affect the efficacy of ICI therapy, in a manner dependent on TMB

**Figure 5:**
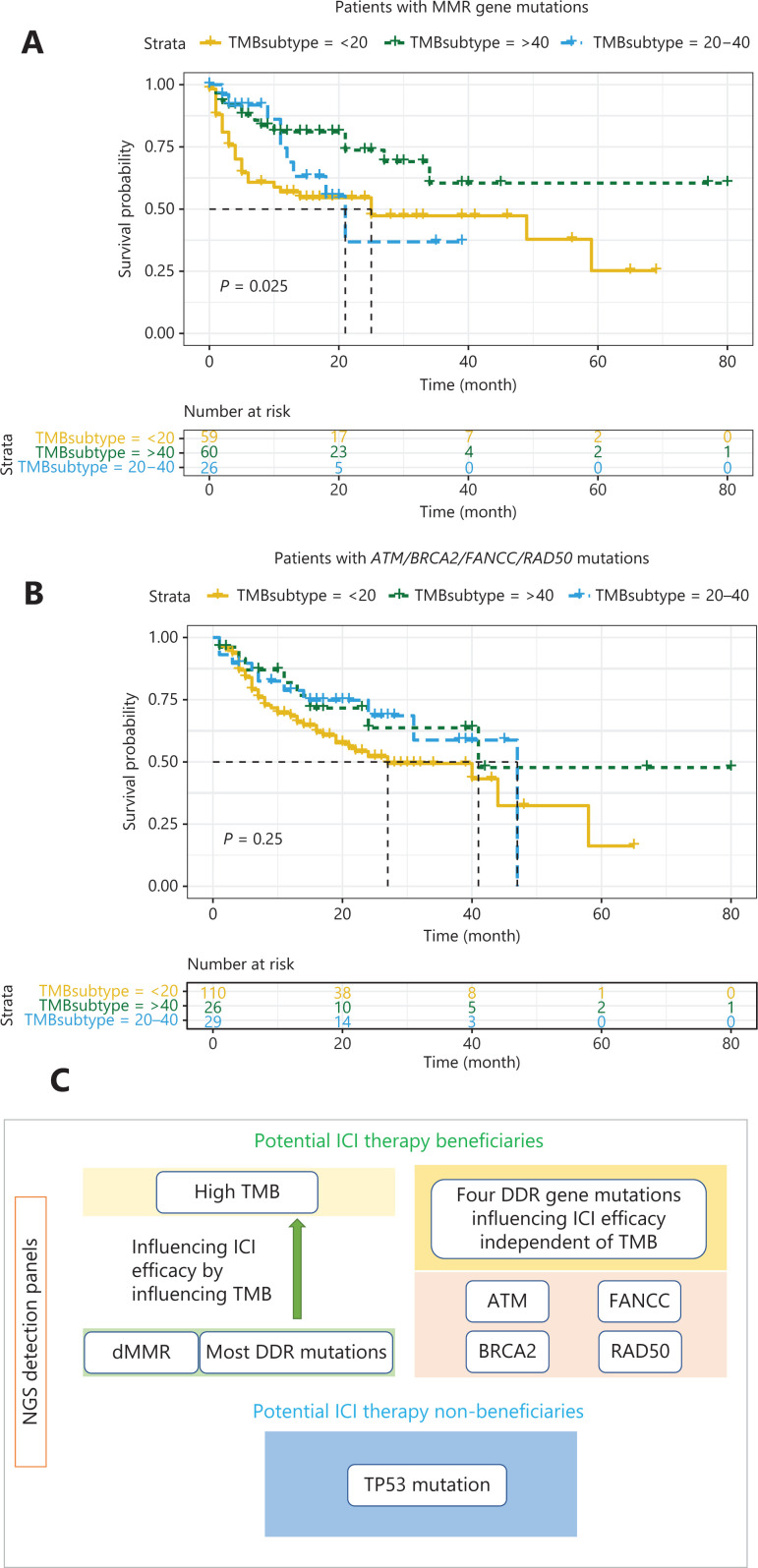
Associations between MMR/DDR mutations and TMB in ICI therapy. (A) Kaplan–Meier analysis of patients with MMR gene mutations and different TMB. (B) Kaplan–Meier analysis of patients with *ATM*/*BRCA2*/*FANCC*/*RAD50* mutations and different TMB. (C) Summary of the workflow for using NGS detection panels to identify potential beneficiaries of ICI therapy.

To further explore the potential mechanism underlying the association between MMR/DDR mutations and the therapeutic efficacy of ICI, we performed univariate Cox regression (**Supplementary Table S5**) in the MSK-IMPACT cohort and found that *ATM*, *BRCA2*, *ERCC4*, *NBN*, *POLE*, *RAD50*, and *TP53* mutations, cancer types, MMR status, and TMB were associated with OS (all *P* < 0.05). Thus, we performed multivariate Cox analyses without TMB (with each DDR gene), and identified *POLE* and *RAD50* mutations as independent prognostic indicators regardless of cancer type and MMR status (**Tables 1 and 2**). However, multivariate Cox regression with TMB, cancer type, and MMR status showed that MMR status, and *POLE* and *RAD50* mutations were no longer independent prognostic indicators, whereas TMB was an independent prognostic indicator (**Tables 1 and 2**). Furthermore, patients with MMR gene mutations and high TMB had better ICI therapy outcomes than patients with MMR gene mutations and low TMB (**Figure 5A**, *P* < 0.05). Moreover, we observed that DDR gene mutations in *ATM*, *BRCA2*, *FANCC*, and *RAD50* were associated with TMB, but the differences in TMB between the mutant and wild-type groups were relatively small, and the median TMB of the mutant group was <15 mutations/MB. However, the differences in outcomes between the mutant and wild-type groups for these 4 DDR genes remained significant. To investigate whether the effects of these 4 DDR gene mutations on the therapeutic efficacy of ICI was dependent on TMB, we combined the 4 DDR gene mutations and performed univariate and multivariate Cox regressions with TMB and cancer type in all patients except those in the MMR mutant subgroup. The 4 combined DDR gene mutations were independent prognostic indicators regardless of cancer type and TMB (**Table 3**). There was no significant difference in ICI therapy outcomes in patients with these 4 DDR gene mutations and different TMBs (**Figure 5B**, *P* = 0.25).

**Table 1:** Multivariate Cox analyses with and without TMB for *POLE* mutations

Factors	Multivariate analysis without TMB		Multivariate analysis with TMB	
	HR (95% CI)	*P*	HR (95% CI)	*P*
Cancer type				
Lung cancer	Ref			
Bladder cancer	0.77 (0.60–0.98)	0.032	0.79 (0.62–1.0)	0.055
Breast cancer	1.4 (0.95–2.0)	0.088	1.3 (0.88–1.9)	0.197
CNS tumor	1.2 (0.91–1.5)	0.218	1.1 (0.87–1.4)	0.410
Esophagogastric cancer	1.1 (0.84–1.5)	0.442	1.1 (0.80–1.4)	0.616
Colorectal cancer	0.82 (0.59–1.1)	0.242	0.87 (0.63–1.2)	0.409
Head and neck cancer	1.1 (0.82–1.4)	0.636	1.0 (0.79–1.3)	0.851
Melanoma	0.40 (0.32–0.51)	<0.001	0.43 (0.34–0.54)	<0.001
Renal cancer	0.35 (0.26–0.47)	<0.001	0.33 (0.25–0.44)	<0.001
Skin cancer, non-melanoma	0.00 (0.00-Inf)	0.927	0.00 (0.00-Inf)	0.930
Primary unknown	1.1 (0.78–1.6)	0.560	1.1 (0.77–1.6)	0.631
MMR status				
pMMR	Ref		Ref	
dMMR	0.73 (0.59–0.91)	0.004	0.95 (0.74–1.2)	0.670
*POLE*				
Wild type	Ref		Ref	
Mutant	0.77 (0.64–0.93)	0.007	0.85 (0.70–1.0)	0.107
TMB			0.99 (0.98–0.99)	<0.001

**Table 2:** Multivariate Cox analyses with and without TMB for *RAD50* mutations

Factors	Multivariate analysis without TMB		Multivariate analysis with TMB	
	HR (95% CI)	*P*	HR (95% CI)	*P*
Cancer type				
Lung cancer	Ref		Ref	
Bladder cancer	0.77 (0.60–0.98)	0.032	0.79 (0.62–1.0)	0.058
Breast cancer	1.4 (0.98–2.0)	0.068	1.3 (0.89–1.9)	0.176
CNS tumor	1.2 (0.91–1.5)	0.210	1.1 (0.86–1.4)	0.422
Esophagogastric cancer	1.1 (0.83–1.5)	0.468	1.1 (0.80–1.4)	0.614
Colorectal cancer	0.83 (0.60–1.1)	0.247	0.88 (0.63–1.2)	0.436
Head and neck cancer	1.1 (0.82–1.4)	0.637	1.0 (0.79–1.3)	0.854
Melanoma	0.40 (0.32–0.50)	<0.001	0.43 (0.34–0.54)	<0.001
Renal cancer	0.35 (0.27–0.47)	<0.001	0.33 (0.25–0.44)	<0.001
Skin cancer, non-melanoma	0.00 (0.00-Inf)	0.927	0.00 (0.00-Inf)	0.930
Primary unknown	1.1 (0.77–1.6)	0.597	1.1 (0.76–1.5)	0.661
MMR status				
pMMR	Ref		Ref	
dMMR	0.72 (0.58–0.88)	0.002	0.95 (0.74–1.2)	0.659
*RAD50*				
Wild type	Ref		Ref	
Mutant	0.65 (0.45–0.94)	0.023	0.73 (0.50–1.1)	0.107
TMB			0.99 (0.98–0.99)	<0.001

**Table 3:** Univariate and multivariate Cox regression in the entire MSK-IMPACT cohort except the MMR mutant subgroup for the combined 4 DDR gene mutations

Factors	Univariate analysis		Multivariate analysis	
	HR (95% CI)	*P*	HR (95% CI)	*P*
Cancer type				
Lung cancer	Ref		Ref	
Bladder cancer	0.77 (0.61–0.99)	0.037	0.82 (0.65–1.1)	0.118
Breast cancer	1.4 (0.97–2.1)	0.071	1.3 (0.88–1.9)	0.202
CNS tumor	1.2 (0.90–1.5)	0.265	1.0 (0.80–1.3)	0.785
Esophagogastric cancer	1.1 (0.83–1.5)	0.465	1.1 (0.80–1.4)	0.652
Colorectal cancer	0.81 (0.58–1.1)	0.240	0.86 (0.61–1.2)	0.405
Head and neck cancer	1.1 (0.83–1.4)	0.600	1.0 (0.77–1.3)	0.998
Melanoma	0.40 (0.32–0.50)	<0.001	0.43 (0.34–0.54)	<0.001
Renal cancer	0.36 (0.27–0.48)	<0.001	0.32 (0.24–0.43)	<0.001
Primary unknown	1.1 (0.78–1.6)	0.559	1.1 (0.75–1.5)	0.704
TMB	0.98 (0.97–0.99)	<0.001	0.98 (0.97–0.99)	<0.001
*ATM*/*BRCA2*/*FANCC*/*RAD50* (wild type)	1.6 (1.3–2.1)	<0.001	1.4 (1.1–1.8)	0.017

### Mutation rates of 6 MMR and 21 DDR genes in diverse cancer types

To investigate the feasibility of using MMR and DDR genes in predicting the efficacy of ICI in diverse cancer types, we analyzed the mutation rates of 6 MMR genes (**Supplementary Table S6**) and 21 DDR genes (including *TP53*) (**Supplementary Table S7**) in 22 cancer types in all 39,631 patients. Although the mutation rate of any given DDR/MMR gene (except *TP53*) was low, the overall mutation rate of DDR/MMR genes was relatively high, reaching 10%–30% in several cancer types.

## Discussion

Currently, the universally recognized biomarkers for predicting the therapeutic efficacy of ICI include PD-1/PD-L1, and MMR/MSI status and TMB. Our findings indicated that patients diagnosed with MSI-H or MMR mutations tended to have higher TMB. We also found that some patients diagnosed with MSI-H had no MMR gene mutations, whereas most of the patients had DDR gene mutations, thus suggesting that DDR gene mutations may also lead to MSI-H. In addition, after excluding MSI-H and MMR gene mutant patients, we found that DDR gene mutations were associated with high TMB, thereby suggesting that DDR gene mutations might result in high TMB.

We also observed that high TMB was associated with better outcomes of ICI therapy and a high absolute score of tumor-infiltrating CD8+ T cells. According to previous studies, high TMB often leads to high neoantigen levels and further activates the immune response of T cells^^22,23^^. Thus, factors that increase TMB might indirectly activate the immune response of T cells, thus further leading to a dense infiltration of lymphocytes and eventually resulting in an active response to ICI treatment.

Clinicians usually use immunohistochemistry to detect the expression of 4 MMR proteins, MLH1, MSH2, MSH6, and PMS2, to determine whether a patient has dMMR or mismatch repair proficient (pMMR) tumors^5,24^. However, immunohistochemistry lacks standardization, and it sometimes returns false positive or false negative results. Our results indicated that by using NGS to detect mutations of 6 MMR genes, *MLH1*, *MSH2*, *MSH3*, *MSH6*, *PMS1*, and *PMS2*, any MMR gene mutation is associated with MSI-H, high TMB, and favorable efficacy of ICI therapy. Although the sequencing method will miss some of the dMMR caused by epigenetics, NGS can detect multiple gene mutations simultaneously, particularly through panels that contain hundreds of genes and can simultaneously detect MMR/DDR gene mutations, MSI status, TMB, and other therapeutic targets, thus making NGS more convenient and practical.

To identify outcomes predictive of DDR gene mutations for ICI treatment, we first excluded patients in the MMR mutant subgroup to avoid the effects of MMR and DDR gene comutations. Mutations in MMR genes in patients in the TMB-L group did not result in high levels of TMB, thus possibly indicating that these mutations are only nonfunctional mutations. Thus, only patients in the TMB-H group with at least one MMR gene mutation were placed in the MMR mutant subgroup, to ensure that patients in the MMR mutant subgroup had functional MMR gene mutations. For the remaining patients in the MSK-IMPACT cohort, 20 DDR gene mutations were significantly associated with efficacy.

We also validated our findings by using the DFCI cohort, and the MMR and DDR gene mutations that we found, except *TP53*, were associated with high ORR but not OS, thus indicating that these gene mutations are predictive rather than prognostic factors for ICI therapy. The small sample size and mixed cancer types in the DFCI cohort might explain why a high ORR did not result in better OS. In addition, the results indicated that *TP53* mutations might be associated with poorer prognosis instead of the therapeutic efficacy of ICI, in agreement with previously reported findings^25–27^. Studies^28,29^ have shown that *TP53* mutants gain oncogenic functions, whereas most DDR gene mutants lose their functions. *TP53* mutants can reprogram macrophages into tumor-supporting macrophages^28^ or promote TGFβ-induced metastasis^29^, thus potentially explaining the poorer prognosis in patients with *TP53* mutations. Therefore, these differences might explain the different behavior of *TP53* mutational status in predicting the therapeutic efficacy of ICI.

Patients with any of the 20 DDR gene mutations had a significantly high median TMB. *POLE* and *RAD50* mutations were identified as independent prognostic indicators regardless of cancer type and MMR status. However, multivariate Cox regression with TMB, cancer types, and MMR status showed that *POLE* and *RAD50* mutations were not independent prognostic indicators, whereas TMB was an independent prognostic indicator. Most DDR gene mutations were associated with ICI treatment efficacy by influencing the TMB. Our study also revealed the inconsistency between MMR mutation status and MSI status, as well as MSI status and TMB; our results indicated that mutations in MMR genes and most DDR genes that result in high TMB affect the efficacy of ICI therapy in a manner dependent on TMB. Thus, although the predictive value of TMB for the therapeutic efficacy of ICI has been controversial^30^, we found that high TMB was associated with better efficacy and was an important independent pan-cancer predictor. Further deeper analysis of combined mutation status is needed and would help strengthen the association with TMB and the therapeutic efficacy of ICI. More importantly, we found that 4 DDR gene mutations were independent prognostic indicators regardless of cancer type and TMB. Therefore, mechanisms may exist that affect the efficacy of ICI therapy other than TMB, and these mechanisms require further exploration. Because these 4 DDR gene mutations predicted the therapeutic efficacy of ICI independently of TMB, they should be included in NGS detection panels. We believe that the 4 DDR gene mutations could aid in identifying more patients who would benefit from ICI therapy and might become a helpful supplement to the current system used to predict the therapeutic efficacy of ICI.

The mutation rates of 6 MMR genes and 21 DDR genes (including *TP53*) in 22 cancer types in all 39,631 patients were also analyzed in our study. Although the mutation rate of any given DDR/MMR gene was low, the overall mutation rate of DDR/MMR genes was relatively high, reaching 10%–30% in several cancer types. These patients are notable beneficiaries of ICI treatment. In addition, these patients are potential beneficiaries of PARP inhibitor treatment^31^. Therefore, the status of multiple MMR and DDR genes in clinical practice must be analyzed, particularly the genes associated with TMB and the efficacy of ICI treatment found in our study. In **Figure 5C**, we summarize the workflow for using NGS detection panels to identify potential beneficiaries of ICI treatment.

This study has some limitations. First, this was a retrospective study; therefore, our conclusions may require further validation from prospective studies. Second, because our study population included diverse cancer types, the relative explanatory roles of the factors analyzed may vary in specific cancer types.

## Conclusions

In summary, our study explored the associations among MMR status, DDR gene mutations, TMB, and the outcomes of ICI treatment across diverse solid tumor types and 20 DDR gene mutations identified to be associated with ICI treatment efficacy. Our results indicated that MMR status and most DDR gene mutations influence the efficacy of ICI treatment by affecting the TMB. Our study revealed that the effects of MMR gene mutations and most DDR gene mutations on the efficacy of ICI therapy depend on TMB, whereas 4 DDR gene mutations are associated with the efficacy of ICI therapy and are not dependent on TMB. Thus, determining only patients’ TMB is insufficient; instead, MMR and DDR genes, particularly the genes identified herein, should additionally be detected in large multigene panels. These genes have predictive value in assessing the efficacy of ICI therapy and thus may provide better guidance for clinical practice and aid in exploration of underlying mechanisms.
