# Supplementary material for: Evaluation of 30 DNA damage response and 6 mismatch repair gene mutations as biomarkers for immunotherapy outcomes across multiple solid tumor types
Source: Cancer Biol Med. 2021 May 7;18(4):1080–91. doi: 10.20892/j.issn.2095-3941.2020.0351 (PMC8610155; doi:10.20892/j.issn.2095-3941.2020.0351)
Supplement: Supplementary file 1 [file cbm-18-1080-s001.pdf]

Supplementary materials

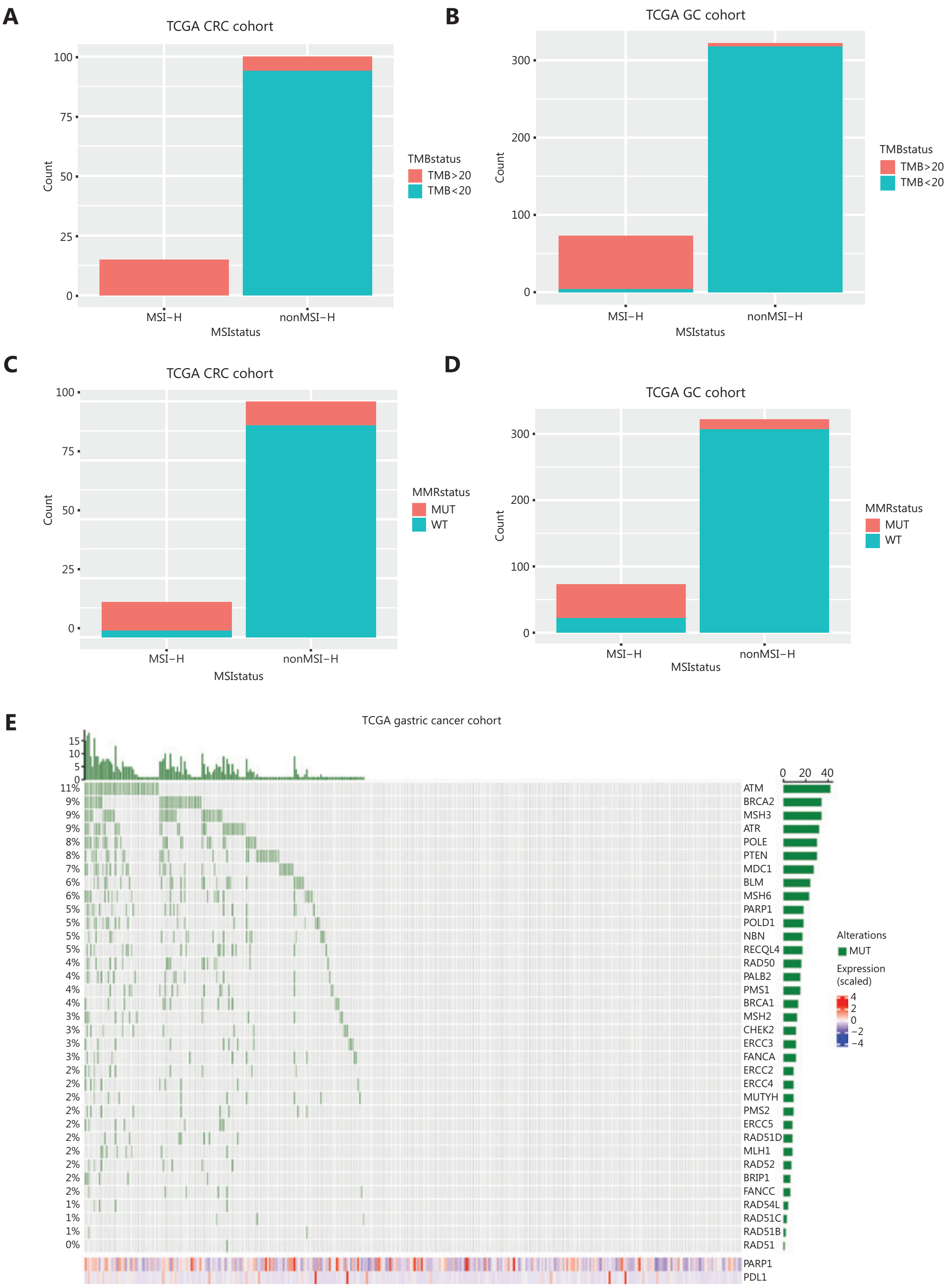

Figure S1 Associations among MSI status, TMB status, DDR gene mutations and the expression of PARP1 and PDL1 in TCGA colorectal and gastric cohorts. Patients diagnosed with MSI-H tended to have high TMB in TCGA colorectal (A) and gastric (B) cohorts. Patients diagnosed with MSI-H tended to have MMR gene mutations in TCGA colorectal (C) and gastric (D) cohorts. (E) Association between the MMR/DDR mutational status and PARP1/PDL1 expression levels.

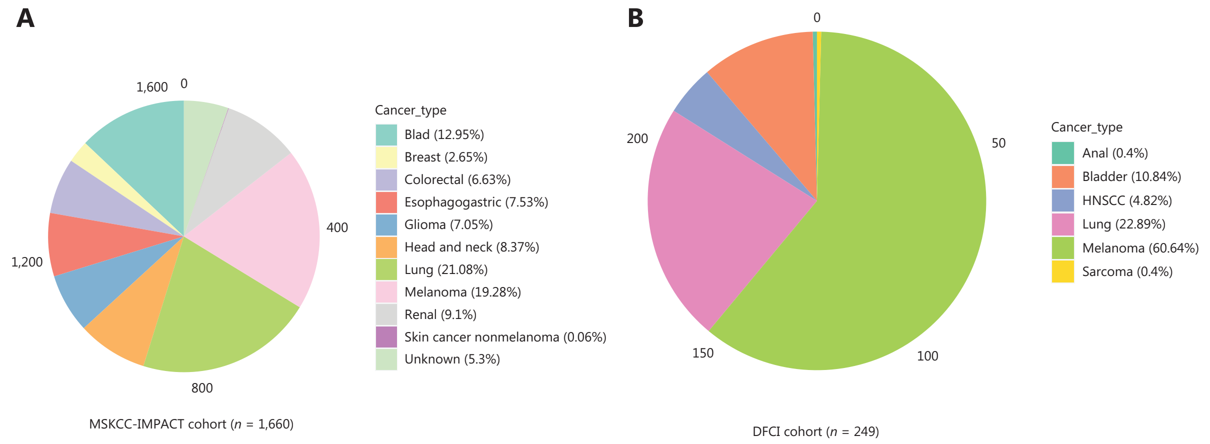

Figure S2 The distribution of different types of cancer in the MSKCC-IMPACT (A) and DFCI (B) cohorts.

Table S1 Univariate Cox regression in the MSK-IMPACT cohort

Factors	Univariate analysis	
	HR (95% CI)	*P*
Cancer type		
Lung cancer	Ref	
Bladder cancer	0.75 (0.59–0.95)	0.018
Breast cancer	1.4 (0.94–2.0)	0.097
CNS tumor	1.2 (0.91–1.5)	0.211
Esophagogastric cancer	1.1 (0.81–1.5)	0.591
Colorectal cancer	0.71 (0.51–0.98)	0.035
Head and neck cancer	1.1 (0.83–1.4)	0.602
Melanoma	0.39 (0.32–0.49)	<0.001
Renal cancer	0.36 (0.27–0.48)	<0.001
Skin cancer, non-melanoma	0.00 (0.00-Inf)	0.919
Primary unknown	1.1 (0.77–1.7)	0.606
MMR status (pMMR)	2.1 (1.4–3.2)	<0.001
TMB	0.98 (0.98–0.99)	<0.001
ATM (wild type)	1.5 (1.1–2.1)	0.009
BRCA2 (wild type)	1.5 (1.1–2.1)	0.015
ERCC4 (wild type)	2.1 (1.1–4.0)	0.028
NBN (wild type)	2.2 (1.1–4.4)	0.026
POLE (wild type)	1.8 (1.2–2.6)	0.002
RAD50 (wild type)	2.6 (1.2–5.4)	0.013
TP53 (wild type)	0.73 (0.63–0.83)	<0.001

Table S2 The median TMB for patients in TCGA colorectal and gastric cancer cohorts with and without MMR gene mutations

Median TMB (mutations/MB) in TCGA colorectal cancer cohort			
HUGO symbol	Mutant	Wild type	*P*
MLH1	63.41 (29.79–449.18)	4.24 (2.13–298.05)	0.034
MSH2	115.46 (29.79–449.18)	4.21 (2.13–201.26)	0.010
MSH3	249.66 (101.05–449.18)	4.24 (2.13–195.00)	0.122
MSH6	82.45 (4.18–449.18)	4.18 (2.13–201.26)	0.010
PMS1	246.53 (33.34–449.18)	4.24 (2.13–201.26)	0.122
PMS2	69.21 (3.26–298.05)	4.22 (2.13–449.18)	0.053
Median TMB (mutations/MB) in TCGA gastric cancer cohort			
HUGO symbol	Mutant	Wild type	*P*
MLH1	39.89 (16.42–56.87)	4.34 (0.18–232.24)	0.011
MSH2	41.79 (3.84–232.24)	4.34 (0.18–227.95)	0.020
MSH3	46.54 (2.84–232.24)	4.08 (0.18–227.95)	<0.001
MSH6	47.92 (2.58–232.24)	4.21 (0.18–227.95)	<0.001
PMS1	46.82 (2.21–135.79)	4.29 (0.18–232.24)	0.001
PMS2	49.63 (23.71–232.24)	4.33 (0.18–227.95)	0.006

Table S3 The median TMB of patients in TCGA colorectal cancer cohort with and without DDR gene mutations

HUGO symbol	Mutated	Non-mutated	*P*
	Median TMB (mutations/MB)	Median TMB (mutations/MB)	
ATM	47.50 (2.53–449.18)	4.17 (2.13–92.71)	0.014
ATR	97.99 (3.39–449.18)	4.21 (2.13–195.00)	0.019
BLM	162.43 (69.21–449.18)	4.24 (2.13–201.26)	0.034
BRCA1	165.57 (75.79–449.18)	4.24 (2.13–195.00)	0.034
BRCA2	92.71 (3.26–449.18)	4.22 (2.13–201.26)	0.035
BRIP1	162.43 (3.32–449.18)	4.24 (2.13–101.05)	0.063
CHEK2	94.92 (94.92–94.92)	4.30 (2.13–449.18)	0.993
ERCC2	63.03 (33.34–92.71)	4.29 (2.13–449.18)	0.468
ERCC3	249.66 (36.87–449.18)	4.24 (2.13–195.00)	0.122
ERCC4	165.57 (69.21–449.18)	4.24 (2.13–298.05)	0.122
ERCC5	143.86 (92.71–195.00)	4.29 (2.13–449.18)	0.468
FANCA	77.33 (2.63–449.18)	4.24 (2.13–201.26)	0.063
FANCC	195.00 (101.05–201.26)	4.26 (2.13–449.18)	0.236
MDC1	195.00 (33.34–449.18)	4.22 (2.13–129.87)	0.018
MUTYH	44.37 (5.16–449.18)	4.24 (2.13–298.05)	0.064
NBN	46.45 (2.68–449.18)	4.29 (2.13–298.05)	0.598
PALB2	47.50 (3.89–449.18)	4.22 (2.13–298.05)	0.035
PARP1	69.21 (4.87–298.05)	4.24 (2.13–449.18)	0.064
POLD1	75.79 (47.50–92.71)	4.26 (2.13–449.18)	0.236
POLE	101.05 (2.63–449.18)	4.20 (2.13–92.71)	0.010
PTEN	94.92 (2.34–449.18)	4.24 (2.13–201.26)	0.157
RAD50	129.87 (3.32–449.18)	4.24 (2.13–201.26)	0.113
RAD51	201.26 (201.26–201.26)	4.30 (2.13–449.18)	0.993
RAD51B	/	4.32 (2.13–449.18)	/
RAD51C	100.42 (5.84–195.00)	4.29 (2.13–449.18)	0.468
RAD51D	165.70 (33.34–298.05)	4.29 (2.13–449.18)	0.468
RAD52	94.92 (5.11–449.18)	4.26 (2.13–298.05)	0.236
RAD54L	58.36 (29.79–129.87)	4.24 (2.13–449.18)	0.122
RECQL4	92.71 (92.71–92.71)	4.30 (2.13–449.18)	0.993
TP53	3.92 (2.13–449.18)	4.86 (2.34–201.26)	0.075

Table S4 The median TMB for patients in TCGA gastric cancer cohort with and without DDR gene mutations

HUGO symbol	Mutated	Non-mutated	*P*
	Median TMB (mutations/MB)	Median TMB (mutations/MB)	
ATM	36.64 (1.87–232.24)	4.18 (0.18–135.79)	<0.001
ATR	37.87 (1.24–232.24)	4.17 (0.18–97.74)	<0.001
BLM	45.04 (3.26–227.95)	4.18 (0.18–232.24)	<0.001
BRCA1	44.29 (3.05–192.71)	4.32 (0.18–232.24)	0.007
BRCA2	44.29 (1.68–232.24)	4.08 (0.18–108.61)	<0.001
BRIP1	86.47 (22.68–232.24)	4.37 (0.18–227.95)	0.036
CHEK2	35.42 (2.58–232.24)	4.36 (0.18–227.95)	0.058
ERCC2	92.00 (33.39–227.95)	4.33 (0.18–232.24)	0.006
ERCC3	41.92 (3.47–192.71)	4.36 (0.18–232.24)	0.058
ERCC4	56.87 (3.87–227.95)	4.39 (0.18–232.24)	0.161
ERCC5	66.08 (30.39–192.71)	4.33 (0.18–232.24)	0.006
FANCA	50.92 (2.34–232.24)	4.32 (0.18–192.71)	0.007
FANCC	48.32 (2.55–135.08)	4.42 (0.18–232.24)	0.201
MDC1	44.29 (1.66–232.24)	4.18 (0.18–135.08)	<0.001
MUTYH	50.68 (3.18–227.95)	4.37 (0.18–232.24)	0.097
NBN	45.42 (1.55–135.08)	4.30 (0.18–232.24)	0.002
PALB2	56.71 (4.89–232.24)	4.26 (0.18–227.95)	<0.001
PARP1	47.74 (11.53–232.24)	4.21 (0.18–192.71)	<0.001
POLD1	45.88 (2.66–232.24)	4.21 (0.18–192.71)	<0.001
POLE	52.54 (3.16–232.24)	4.16 (0.18–95.29)	<0.001
PTEN	38.42 (2.18–227.95)	4.22 (0.18–227.95)	0.022
RAD50	46.63 (2.68–232.24)	4.29 (0.18–227.95)	0.001
RAD51	50.21 (50.21–50.21)	4.46 (0.18–232.24)	0.986
RAD51B	92.36 (49.63–135.08)	4.45 (0.18–232.24)	0.463
RAD51C	9.68 (3.79–66.66)	4.46 (0.18–232.24)	0.975
RAD51D	49.33 (4.32–108.61)	4.37 (0.18–232.24)	0.066
RAD52	59.79 (41.79–227.95)	4.34 (0.18–232.24)	0.011
RAD54L	79.41 (37.87–227.95)	4.42 (0.18–232.24)	0.123
RECQL4	45.92 (4.47–232.24)	4.21 (0.18–192.71)	<0.001
TP53	4.72 (0.71–232.24)	3.71 (0.18–227.95)	0.008

Table S5 Associations between MMR/DDR mutations and TMB in the MSK-IMPACT and DFCI cohorts

HUGO symbol	MSK-IMPACT cohort					
	All patients			All patients except dMMR group		
	Mutated	Non-mutated	*P*	Mutated	Non-mutated	*P*
	Median TMB (mutations/MB)	Median TMB (mutations/MB)		Median TMB (mutations/MB)	Median TMB (mutations/MB)	
ATM	17.71 (2.63–209.55)	5.90 (0.00–163.31)	<0.001	11.81 (2.63–150.52)	5.58 (0.00–163.31)	<0.001
ATR	24.58 (2.95–209.55)	5.90 (0.00–181.78)	<0.001	17.55 (2.95–150.52)	5.58 (0.00–163.31)	<0.001
BLM	34.44 (3.94–209.55)	5.90 (0.00–181.78)	<0.001	28.8 (3.94–163.31)	5.58 (0.00–126.02)	<0.001
BRCA1	29.51 (1.76–209.55)	5.90 (0.00–203.64)	<0.001	17.63 (1.76–163.31)	5.58 (0–150.52)	<0.001
BRCA2	26.11 (1.97–209.55)	5.90 (0.00–112.35)	<0.001	14.76 (1.97–163.31)	5.58 (0.00–100.06)	<0.001
BRIP1	23.61 (6.69–203.64)	5.90 (0.00–209.55)	<0.001	19.68 (6.69–126.02)	5.58 (0.00–163.31)	<0.001
CHEK2	36.95 (4.39–209.55)	5.90 (0.00–203.64)	<0.001	32.48 (4.39–66.90)	5.90 (0.00–163.31)	0.003
ERCC2	21.15 (3.94–209.55)	5.90 (0.00–203.64)	<0.001	19.31 (3.94–163.31)	5.90 (0.00–126.02)	<0.001
ERCC3	48.82 (9.84–203.64)	5.90 (0.00–209.55)	<0.001	24.59 (9.84–163.31)	5.90 (0.00–150.52)	0.005
ERCC4	35.69 (6.89–209.55)	5.90 (0.00–203.64)	<0.001	23.61 (6.89–100.06)	5.90 (0.00–163.31)	<0.001
ERCC5	28.96 (1.97–209.55)	5.90 (0.00–181.78)	<0.001	18.26 (1.97–163.31)	5.90 (0.00–150.52)	0.003
FANCA	26.07 (3.35–209.55)	5.90 (0.00–203.64)	<0.001	17.55 (3.35–59.03)	5.90 (0.00–163.31)	<0.001
FANCC	38.79 (4.39–178.44)	5.90 (0.00–209.55)	<0.001	14.76 (4.39–66.90)	5.90 (0.00–163.31)	0.09
MDC1	26.33 (3.35–209.55)	5.90 (0.00–203.64)	<0.001	17.71 (3.35–163.31)	5.90 (0.00–100.06)	<0.001
MLH1	30.12 (2.63–153.47)	5.90 (0.00–209.55)	<0.001	/	5.90 (0.00–163.31)	/
MSH2	44.27 (4.92–209.55)	5.90 (0.00–203.64)	<0.001	/	5.90 (0.00–163.31)	/
MSH3	58.81 (9.65–112.35)	5.90 (0.00–209.55)	<0.001	/	5.90 (0.00–163.31)	/
MSH6	51.16 (2.23–209.55)	5.90 (0.00–163.31)	<0.001	/	5.90 (0.00–163.31)	/
MUTYH	31.33 (4.46–203.64)	5.90 (0.00–209.55)	0.002	14.50 (4.46–150.52)	5.90 (0.00–163.31)	0.054
NBN	34.57 (3.94–209.55)	5.90 (0.00–178.44)	<0.001	24.09 (3.94–69.85)	5.90 (0.00–163.31)	0.001
PALB2	24.10 (3.94–209.55)	5.90 (0.00–203.64)	<0.001	19.32 (3.94–68.87)	5.90 (0.00–163.31)	<0.001
PARP1	33.07 (6.14–181.78)	5.90 (0.00–209.55)	<0.001	21.94 (6.14–163.31)	5.90 (0.00–150.52)	<0.001
PMS1	44.76 (5.90–203.64)	5.90 (0.00–209.55)	<0.001	/	5.90 (0.00–163.31)	/
PMS2	14.29 (3.94–209.55)	5.90 (0.00–203.64)	0.005	/	5.90 (0.00–163.31)	/
POLD1	42.30 (3.94–112.35)	5.90 (0.00–209.55)	<0.001	16.23 (3.94–66.90)	5.90 (0.00–163.31)	0.001
POLE	28.75 (2.63–209.55)	5.90 (0.00–181.78)	<0.001	17.71 (2.63–163.31)	5.90 (0.00–126.02)	<0.001
PTEN	6.89 (1.76–181.78)	5.90 (0.00–209.55)	0.058	6.69 (1.76–163.31)	5.90 (0.00–126.02)	0.336
RAD50	47.22 (1.12–209.55)	5.90 (0.00–181.78)	<0.001	14.76 (1.12–126.02)	5.90 (0.00–163.31)	0.003
RAD51	27.21 (6.89–52.14)	5.90 (0.00–209.55)	0.007	25.65 (6.89–46.52)	5.90 (0.00–163.31)	0.018
RAD51B	24.10 (6.14–102.31)	5.90 (0.00–209.55)	<0.001	20.66 (6.14–47.22)	5.90 (0.00–163.31)	<0.001
RAD51C	40.15 (14.76–181.78)	5.90 (0.00–209.55)	0.002	35.00 (14.76–69.85)	5.90 (0.00–163.31)	0.01

Table S5 Continued

HUGO symbol	MSK-IMPACT cohort					
	All patients			All patients except dMMR group		
	Mutated	Non-mutated	*P*	Mutated	Non-mutated	*P*
	Median TMB (mutations/MB)	Median TMB (mutations/MB)		Median TMB (mutations/MB)	Median TMB (mutations/MB)	
RAD51D	36.66 (3.94–181.78)	5.90 (0.00–209.55)	0.67	13.28 (3.94–52.66)	5.90 (0.00–163.31)	0.569
RAD52	49.37 (13.38–178.44)	5.90 (0.00–209.55)	0.004	27.21 (13.38–65.91)	5.90 (0.00–163.31)	0.062
RAD54L	54.11 (9.84–209.55)	5.90 (0.00–181.78)	0.001	30.13 (9.84–95.43)	5.90 (0.00–163.31)	0.033
RECQL4	36.67 (2.95–181.78)	5.90 (0.00–209.55)	<0.001	14.04 (2.95–66.90)	5.90 (0.00–163.31)	<0.001
TP53	7.87 (0.88–209.55)	4.92 (0.00–153.47)	<0.001	6.89 (0.88–163.31)	4.92 (0.00–68.87)	<0.001

HUGO symbol	DFCI cohort					
	All patients			All patients except dMMR group		
	Mutated	Non-mutated	*P*	Mutated	Non-mutated	*P*
	Median TMB (mutations/MB)	Median TMB (mutations/MB)		Median TMB (mutations/MB)	Median TMB (mutations/MB)	
ATM	18.84 (1.99–305.11)	8.95 (0.55–93.53)	0.030	11.75 (1.99–48.71)	7.93 (0.55–84.61)	0.241
ATR	35.1 (4.17–305.11)	8.75 (0.55–93.53)	0.001	13.27 (4.17–84.61)	7.93 (0.55–80.07)	0.069
BLM	25.22 (3.21–255.59)	9.26 (0.55–305.11)	0.212	14.24 (3.21–34.84)	8.25 (0.55–84.61)	0.442
BRCA1	31.76 (2.76–305.11)	8.93 (0.55–146.29)	0.011	12.51 (2.76–80.07)	8.03 (0.55–84.61)	0.195
BRCA2	40.41 (5.96–305.11)	8.65 (0.55–119.1)	<0.001	16.83 (5.96–50.77)	7.78 (0.55–84.61)	0.008
BRIP1	37.43 (3.74–305.11)	9.26 (0.55–146.29)	0.106	14.92 (3.74–49.89)	8.43 (0.55–84.61)	0.719
CHEK2	23.86 (5.96–255.59)	9.7 (0.55–305.11)	0.361	19.06 (5.96–23.86)	8.36 (0.55–84.61)	1.000
ERCC2	20.13 (7.63–255.59)	9.53 (0.55–305.11)	0.276	18.09 (7.63–49.89)	8.25 (0.55–84.61)	0.442
ERCC3	8.92 (1.11–255.59)	9.97 (0.55–305.11)	0.989	8.62 (1.11–41.62)	8.36 (0.55–84.61)	1.000
ERCC4	16.44 (6.04–161.57)	9.87 (0.55–305.11)	0.711	10.09 (6.04–37.43)	8.12 (0.55–84.61)	0.125
ERCC5	30.17 (4.88–255.59)	9.87 (0.55–305.11)	0.609	20.72 (4.88–36.56)	8.43 (0.55–84.61)	0.478
FANCA	28.56 (5.27–161.57)	8.87 (0.55–305.11)	0.001	20.08 (5.27–84.61)	7.78 (0.55–80.07)	0.008
FANCC	132.69 (4.39–255.59)	9.53 (0.55–305.11)	0.211	26.55 (4.39–48.71)	8.43 (0.55–84.61)	0.478
MDC1	43.66 (4.71–305.11)	9.28 (0.55–93.53)	0.164	9.99 (4.71–49.89)	8.19 (0.55–84.61)	0.280
MLH1	45.24 (13.03–255.59)	9.26 (0.55–305.11)	0.021	/	8.31 (0.55–84.61)	/
MSH2	30.6 (5.11–255.59)	9.1 (0.55–305.11)	0.007	/	8.25 (0.55–84.61)	/
MSH3	19.06 (2.75–161.57)	9.41 (0.55–305.11)	0.248	/	8.51 (0.55–84.61)	/
MSH6	75.61 (8.62–305.11)	9.07 (0.55–93.53)	0.004	/	8.25 (0.55–84.61)	/
MUTYH	72.09 (4.96–255.59)	9.26 (0.55–305.11)	0.040	19.06 (4.96–84.61)	8.25 (0.55–80.07)	0.366
NBN	26.87 (10.83–161.57)	9.28 (0.55–305.11)	0.038	14.25 (10.83–37.43)	8.19 (0.55–84.61)	0.130
PALB2	43.27 (3.99–305.11)	9.23 (0.55–255.59)	0.104	10.45 (3.99–21.87)	8.31 (0.55–84.61)	0.614

Table S5 Continued

HUGO symbol	DFCI cohort					
	All patients			All patients except dMMR group		
	Mutated	Non-mutated	*P*	Mutated	Non-mutated	*P*
	Median TMB (mutations/MB)	Median TMB (mutations/MB)		Median TMB (mutations/MB)	Median TMB (mutations/MB)	
PARP1	34.84 (7.33–161.57)	9.26 (0.55–305.11)	0.040	13.32 (7.33–34.84)	8.25 (0.55–84.61)	0.366
PMS1	61.65 (10.83–305.11)	9.26 (0.55–146.29)	0.021	/	8.36 (0.55–84.61)	/
PMS2	54.78 (3.74–255.59)	9.16 (0.55–305.11)	0.032	/	8.51 (0.55–84.61)	/
POLD1	48.59 (2.81–255.59)	9.26 (0.55–305.11)	0.040	10.6 (2.81–48.59)	8.36 (0.55–84.61)	1.000
POLE	30.69 (5.88–255.59)	9.23 (0.55–305.11)	0.005	28.56 (5.88–84.61)	7.92 (0.55–80.07)	0.013
PTEN	13.37 (1–255.59)	9.41 (0.55–305.11)	0.387	10.09 (1.00–49.89)	8.25 (0.55–84.61)	0.647
RAD50	38.36 (1.99–161.57)	9.28 (0.55–305.11)	0.071	20.35 (1.99–41.62)	8.31 (0.55–84.61)	0.614
RAD51	45.53 (19.67–47.92)	9.7 (0.55–305.11)	0.243	33.8 (19.67–47.92)	8.31 (0.55–84.61)	0.478
RAD51B	54.06 (12.49–146.29)	9.41 (0.55–305.11)	0.069	12.49 (12.49–12.49)	8.36 (0.55–84.61)	1.000
RAD51C	65.8 (3.99–161.57)	9.87 (0.55–305.11)	0.609	11.06 (3.99–18.13)	8.43 (0.55–84.61)	0.478
RAD51D	19.33 (5.27–119.1)	9.7 (0.55–305.11)	0.361	12.64 (5.27–19.33)	8.36 (0.55–84.61)	1.000
RAD52	45.69 (4.71–161.57)	9.88 (0.55–305.11)	0.993	7.19 (4.71–45.69)	8.51 (0.55–84.61)	1.000
RAD54L	32.81 (2.61–305.11)	9.7 (0.55–255.59)	0.437	11.03 (2.61–32.81)	8.43 (0.55–84.61)	0.614
RECQL4	20.92 (2.75–255.59)	9.1 (0.55–305.11)	0.019	14.08 (2.75–25.22)	8.03 (0.55–84.61)	0.106
TP53	11.45 (1.23–305.11)	8.93 (0.55–161.57)	0.094	10.05 (1.23–84.61)	8.12 (0.55–80.07)	0.770

Table S6 Mutation rates of 6 MMR genes across 22 cancer types

Cancer type	*MLH1*	*MSH2*	*MSH3*	*MSH6*	*PMS1*	*PMS2*	MMR mutant
Adrenocortical carcinoma	2.56%	2.56%	0.00%	1.71%	0.85%	3.42%	8.55%
Bladder cancer	2.16%	2.92%	1.11%	2.64%	1.39%	1.88%	10.58%
Breast cancer	0.37%	0.31%	0.25%	0.55%	0.50%	0.35%	2.10%
Cervical cancer	2.93%	1.17%	1.17%	1.76%	2.05%	1.17%	8.50%
Cholangiocarcinoma	0.34%	0.68%	0.00%	0.34%	0.68%	0.00%	2.04%
CNS tumor	1.09%	1.33%	0.16%	1.64%	0.70%	0.31%	3.05%
Germ cell tumor	0.55%	0.14%	0.14%	0.68%	0.00%	0.68%	2.05%
Hepatobiliary cancer	0.55%	0.76%	0.62%	1.52%	1.11%	0.42%	4.50%
Ovarian cancer	1.05%	1.05%	0.40%	0.79%	0.79%	0.53%	4.08%
Pancreatic cancer	0.27%	0.55%	0.27%	0.48%	0.27%	0.27%	1.71%
Esophagogastric cancer	1.15%	1.48%	2.88%	2.84%	1.98%	2.02%	9.64%
Colorectal cancer	2.59%	2.89%	1.40%	3.83%	2.34%	1.98%	10.38%
Endometrial cancer	6.33%	6.22%	5.58%	8.37%	4.51%	5.26%	17.27%
Head and neck cancer	0.96%	1.50%	0.86%	0.75%	1.82%	1.82%	6.97%
Lung cancer	1.31%	1.73%	1.33%	1.66%	2.09%	1.54%	8.21%
Melanoma	3.03%	2.97%	3.25%	3.88%	3.31%	3.77%	15.53%
Prostate cancer	0.85%	0.82%	0.82%	0.98%	0.85%	0.56%	3.87%
Renal cancer	1.10%	0.52%	1.04%	0.93%	0.41%	0.58%	4.29%
Sarcoma	0.73%	0.58%	0.80%	0.58%	0.58%	0.29%	2.98%
Skin cancer nonmelanoma	5.02%	3.86%	2.32%	7.72%	5.02%	4.63%	20.46%
Thyroid cancer	0.24%	0.60%	0.12%	0.60%	0.12%	0.12%	1.67%
Unknown	2.13%	1.82%	0.30%	1.82%	0.61%	0.61%	5.78%
ALL	1.31%	1.41%	1.12%	1.78%	1.35%	1.20%	6.23%

Table S7 Mutation rates of 21 DDR genes across 22 cancer types

Cancer type	*ATM*	*ATR*	*BLM*	*BRCA2*	*BRIP1*	*CHEK2*
Adrenocortical carcinoma	5.98%	1.71%	0.00%	0.85%	0.00%	1.71%
Bladder cancer	10.30%	6.54%	3.27%	7.59%	2.85%	2.92%
Breast cancer	2.14%	2.90%	0.73%	2.90%	1.21%	0.72%
Cervical cancer	3.81%	3.81%	2.64%	4.40%	2.93%	1.76%
Cholangiocarcinoma	6.12%	1.36%	0.34%	1.02%	0.34%	0.34%
CNS tumor	1.72%	1.80%	0.63%	2.03%	0.94%	0.39%
Germ cell tumor	0.82%	0.68%	0.68%	0.41%	0.68%	0.68%
Hepatobiliary cancer	4.71%	2.22%	1.04%	2.22%	1.18%	0.62%
Ovarian cancer	1.58%	1.19%	0.79%	2.37%	0.79%	0.66%
Pancreatic cancer	3.29%	0.75%	0.27%	2.05%	0.48%	0.27%
Esophagogastric cancer	6.55%	4.70%	2.51%	5.60%	1.57%	1.44%
Colorectal cancer	8.91%	4.34%	2.54%	6.40%	2.28%	2.13%
Endometrial cancer	15.67%	9.44%	5.90%	12.12%	8.37%	5.15%
Head and neck cancer	3.22%	4.39%	1.93%	4.07%	1.71%	1.07%
Lung cancer	7.95%	4.42%	1.89%	4.76%	2.86%	1.42%
Melanoma	9.08%	8.73%	3.94%	10.22%	4.39%	1.88%
Prostate cancer	3.82%	1.38%	0.53%	3.89%	0.71%	1.20%
Renal cancer	2.72%	1.22%	0.58%	1.22%	0.46%	0.93%
Sarcoma	2.83%	1.60%	0.73%	2.18%	0.80%	0.51%
Skin cancer nonmelanoma	15.83%	14.29%	5.79%	14.29%	4.25%	3.86%
Thyroid cancer	3.10%	0.72%	0.24%	0.60%	0.12%	0.60%
Primary unknown	4.86%	4.86%	2.74%	3.04%	0.91%	1.52%
ALL	5.30%	3.46%	1.57%	4.28%	1.77%	1.30%

–	*ERCC2*	*ERCC3*	*ERCC4*	*ERCC5*	*FANCA*	*FANCC*
Adrenocortical carcinoma	0.00%	0.85%	0.85%	0.85%	0.00%	0.00%
Bladder cancer	9.19%	1.60%	1.39%	1.18%	4.24%	1.25%
Breast cancer	0.39%	0.39%	0.52%	0.45%	1.59%	0.38%
Cervical cancer	2.35%	1.76%	2.05%	1.17%	4.40%	1.17%
Cholangiocarcinoma	0.34%	1.02%	0.34%	0.00%	0.68%	0.34%
CNS tumor	0.78%	0.70%	0.70%	0.55%	1.09%	0.47%
Germ cell tumor	0.41%	0.27%	0.68%	0.00%	0.82%	0.00%
Hepatobiliary cancer	1.45%	0.76%	0.83%	0.97%	1.04%	0.21%
Ovarian cancer	0.40%	0.79%	0.92%	0.79%	1.05%	0.40%

Table S7 Continued

–	*ERCC2*	*ERCC3*	*ERCC4*	*ERCC5*	*FANCA*	*FANCC*
Pancreatic cancer	0.41%	0.55%	0.34%	0.27%	0.82%	0.27%
Esophagogastric cancer	1.40%	1.52%	1.65%	1.24%	2.10%	0.87%
Colorectal cancer	2.26%	2.41%	2.18%	2.16%	3.68%	1.57%
Endometrial cancer	3.76%	4.08%	7.30%	3.86%	6.97%	3.33%
Head and neck cancer	1.39%	0.75%	0.75%	0.86%	2.04%	0.64%
Lung cancer	1.62%	0.97%	1.91%	1.95%	2.12%	0.86%
Melanoma	4.11%	3.42%	4.57%	3.37%	4.91%	2.05%
Prostate cancer	0.82%	0.47%	0.20%	0.73%	0.67%	0.67%
Renal cancer	0.52%	0.29%	0.35%	0.99%	0.64%	0.35%
Sarcoma	0.80%	0.29%	0.36%	0.58%	0.51%	0.65%
Skin cancer nonmelanoma	3.86%	6.18%	5.41%	3.86%	10.42%	1.93%
Thyroid cancer	0.12%	0.12%	0.72%	0.24%	0.48%	0.36%
Primary unknown	0.30%	1.22%	0.91%	0.61%	2.13%	0.00%
ALL	1.54%	1.10%	1.33%	1.20%	2.06%	0.81%

Cancer type	*NBN*	*PARP1*	*POLD1*	*POLE*	*RAD50*	*RAD51*
Adrenocortical carcinoma	0.00%	2.56%	1.71%	1.71%	2.56%	0.85%
Bladder cancer	1.74%	2.09%	2.71%	5.36%	2.85%	1.11%
Breast cancer	0.35%	0.80%	0.45%	1.04%	0.71%	0.26%
Cervical cancer	1.17%	2.35%	1.76%	3.52%	2.35%	0.59%
Cholangiocarcinoma	0.68%	0.34%	0.00%	1.70%	1.02%	0.00%
CNS tumor	0.70%	0.78%	0.94%	1.25%	1.17%	0.08%
Germ cell tumor	0.41%	0.00%	0.41%	0.96%	0.41%	0.14%
Hepatobiliary cancer	0.69%	0.76%	0.55%	1.80%	0.97%	0.14%
Ovarian cancer	0.53%	0.79%	0.92%	0.53%	0.92%	0.00%
Pancreatic cancer	0.27%	0.55%	0.68%	1.16%	0.27%	0.07%
Esophagogastric cancer	2.39%	2.47%	2.55%	4.90%	2.39%	0.25%
Colorectal cancer	2.16%	2.39%	3.78%	5.86%	2.77%	0.41%
Endometrial cancer	5.69%	6.22%	7.08%	11.80%	7.30%	1.93%
Head and neck cancer	1.39%	0.75%	1.93%	2.89%	1.93%	0.54%
Lung cancer	1.64%	1.81%	1.62%	5.13%	1.83%	0.47%
Melanoma	2.57%	3.94%	4.51%	9.70%	3.54%	1.08%
Prostate cancer	0.89%	0.73%	0.76%	1.20%	0.58%	0.18%
Renal cancer	0.58%	0.58%	0.93%	1.04%	0.75%	0.23%

Table S7 Continued

Cancer type	*NBN*	*PARP1*	*POLD1*	*POLE*	*RAD50*	*RAD51*
Sarcoma	0.29%	0.58%	1.16%	2.11%	0.80%	0.15%
Skin cancer nonmelanoma	3.47%	7.72%	7.72%	5.41%	3.86%	0.77%
Thyroid cancer	0.24%	0.12%	0.36%	0.95%	0.24%	0.12%
Primary unknown	0.91%	2.13%	2.74%	3.04%	2.13%	0.30%
ALL	1.25%	1.51%	1.71%	3.28%	1.59%	0.38%

Cancer type	*RAD51B*	*RAD51C*	*TP53*	DDR mutant (except *TP53*)	MMR/DDR mutant
Adrenocortical carcinoma	0.00%	0.85%	23.93%	16.24%	17.95%
Bladder cancer	0.84%	1.39%	44.61%	38.00%	40.50%
Breast cancer	0.13%	0.26%	35.95%	14.43%	15.62%
Cervical cancer	0.59%	1.76%	9.97%	26.98%	30.50%
Cholangiocarcinoma	0.00%	0.34%	22.11%	13.61%	14.97%
CNS tumor	0.39%	0.55%	40.23%	9.61%	10.39%
Germ cell tumor	0.00%	0.00%	7.25%	8.48%	10.26%
Hepatobiliary cancer	0.28%	0.35%	27.70%	20.78%	21.05%
Ovarian cancer	0.40%	0.00%	70.09%	11.99%	15.02%
Pancreatic cancer	0.00%	0.07%	52.12%	10.55%	11.78%
Esophagogastric cancer	0.37%	0.41%	57.44%	25.42%	28.51%
Colorectal cancer	0.66%	0.84%	63.81%	28.25%	30.51%
Endometrial cancer	3.65%	2.36%	45.39%	35.73%	37.45%
Head and neck cancer	0.32%	0.75%	60.77%	24.97%	28.94%
Lung cancer	0.92%	0.78%	62.13%	31.49%	35.39%
Melanoma	2.91%	1.77%	16.61%	42.12%	45.95%
Prostate cancer	0.76%	0.16%	22.77%	14.68%	16.72%
Renal cancer	0.29%	0.06%	7.48%	12.70%	15.42%
Sarcoma	0.44%	0.15%	27.83%	11.77%	13.30%
Skin cancer nonmelanoma	2.70%	2.70%	54.83%	47.49%	49.42%
Thyroid cancer	0.24%	0.12%	8.59%	6.92%	7.76%
Primary unknown	0.00%	0.00%	41.95%	19.15%	21.28%
ALL	0.66%	0.56%	40.47%	21.35%	23.56%
